# Evaluation of recombinant multi-epitope proteins for diagnosis of goat schistosomiasis by enzyme-linked immunosorbent assay

**DOI:** 10.1186/s13071-016-1418-4

**Published:** 2016-03-09

**Authors:** Chao Lv, Yang Hong, Zhiqiang Fu, Ke Lu, Xiaodan Cao, Tao Wang, Chuangang Zhu, Hao Li, Rui Xu, Bingguang Jia, Qian Han, Xuefeng Dou, Yuanxi Shen, Zuhang Zhang, Jinli Zai, Jintao Feng, Jiaojiao Lin

**Affiliations:** Key Laboratory of Animal Parasitology, Ministry of Agriculture of China, Shanghai Veterinary Research Institute, Chinese Academy of Agricultural Sciences, Shanghai, China; College of Life and Environmental Science, Shanghai Normal University, Shanghai, China; Jiangsu Co-innovation Center for Prevention and Control of Important Animal Infectious Diseases and Zoonoses, Yangzhou, China

**Keywords:** Recombinant multi-epitope proteins, Diagnosis, Goat schistosomiasis, ELISA

## Abstract

**Background:**

Schistosomiasis is a huge threat to human and animal health. Apart from bovines, goats play an important role in the transmission of schistosomiasis in some endemic areas of China. An accessible, quality-assured goat schistosomiasis diagnostic technique is needed. Recently, our laboratory identified two recombinant diagnostic antigens, SjPGM and SjRAD23 *via* an immuno-proteomic method. The application of these two recombinant antigens to develop a higher sensitivity and specificity technique for the sheep schistosomiasis diagnosis is urgently needed.

**Methods:**

Epitopes of SjPGM and SjRAD23 were predicted and three polypeptides, two from SjRAD23 and one from SjPGM, were selected. Recombinant plasmids containing two to three DNA sequences encoding predicted polypeptides or large hydrophilic region of Sj23 (LHD-Sj23) were constructed and expressed. Eight recombinant schistosome antigens including four multi-epitope proteins and four recombinant single-molecule antigens as well as SEA, were assessed by ELISA in 91 sera from schistosome-infected goats, 44 sera from non-infected goats, 37 sera from *Orientobilharzia*-infected goats, and 12 from *Haemonchus contortus*-infected goats.

**Results:**

ELISA tests showed that three multi-epitope proteins had higher sensitivity than the four single-molecule antigens (rSjRAD23, rSjPGM, rBSjRAD23-1, rBSj23) and the multi-epitope protein rBSjPGM-BSjRAD23-1-BSj23 had the highest sensitivity (97.8 %, 89/91) and maintained good specificity (100 %, 44/44) as well as low cross-reactivity with haemonchosis (8.33 %, 3/12) and orientobilharziasis (13.51 %, 5/37) in the diagnosis of goat schistosomiasis. In contrast, when SEA was applied as a diagnosis antigen, it had 100 % (91/91) sensitivity, 75 % (33/44) specificity, 25 and 83.78 % cross-reactivity with haemonchosis (3/12) and orientobilharziasis (31/37), respectively.

**Conclusions:**

The application of recombinant multi-epitope proteins may increase the sensitivity of diagnosis technique and retain high specificity of single-molecule antigens for schistosomiasis, and the recombinant antigen rBSjPGM-BSjRAD23-1-BSj23 has the potential to be used as a diagnosis antigen for goat schistosomiasis.

## Background

Schistosomiasis japonica is a major parasitic zoonosis in China and Southeast Asia, which seriously threatens human and animal health in endemic areas. At the end of 2013, there were still 184,943 cases of schistosomiasis, including 29,796 cases of advanced schistosomiasis and 1700 fatal cases in China [1]. Although *Schistosoma japonicum* is known to infect more than 40 species of mammals in addition to humans, schistosome-infected bovines are considered to be the main host reservoir in China; however, in some marshland endemic areas goats are also reported to be a major infection source [2, 3]. There are 1,033,056 cattle and buffaloes, 2,024,512 goats and sheep and 891,301 other domestic animals, estimated to breed in schistosomiasis endemic regions in China in 2012 [4]. A survey from 2005 to 2010 in the Dongting lake region indicated that the proportion of the schistosome-infected bovines was 23.5–58.2 % of total infected ruminants, while there were 41.8–76.5 % infected goats and infection rates in cattle and goats were significantly higher than those found in water buffaloes in the survey years [5]. The studies mentioned above showed both bovines and goats play significant roles in disease transmission in some endemic areas of China. Great achievements in domestic animal schistosomiasis control have been attained over the past six decades in China, the prevalence and intensity of schistosome infection have significantly decreased both in humans and domestic animals [6]. The development of a more sensitive and specific diagnostic technique is urgently required in the low endemic areas for case detection, surveillance and screening of target individuals for treatment [7].

Although the pathogen detection techniques that include stool egg examination and miracidium hatching are the gold standard methods to diagnose domestic animal schistosomiasis, serological diagnosis has its advantage with high sensitivity and easy operation, and has been widely applied in disease diagnosis.

Nowadays, schistosome soluble egg antigen (SEA) has been employed as the diagnosis antigen in most developed serological detecting methods. When applied in detection, SEA usually possesses higher sensitivity than other native parasite antigens, such as adult worm antigen or cercariae antigen, and at the same time has lower specificity, higher cross-reactivity with other parasite infections, and specific antibodies drop slowly post chemotherapy. For increasing the specificity of the diagnosis method, screening of single molecules with potential as diagnosis antigen has been carried out *via* enzyme-linked immunosorbent assay (ELISA), chip technology, pull-down, immune-proteomics and other techniques. Several defined antigens have been identified, their recombinant proteins have been prepared and the effects of these proteins as diagnostic molecules have been evaluated. Generally speaking, most of the purified recombinant single-molecule antigens have shown higher specificity, lower cross-reactivity, but poor sensitivity when compared with those of SEA. Recently, our laboratory has identified two recombinant proteins, rSjPGM (phosphoglycerate mutase) and rSjRAD23 (radiation-sensitive protein) which possess value for schistosomiasis diagnosis by immunoproteomics technology. Our previous studies also showed that rSjPGM and rSjRAD23 were promising diagnostic antigens for water buffalo schistosomiasis [8].

It is speculated that a good way to improve the sensitivity of the diagnostic method may be by constructing and applying multi-epitope recombinant antigens as a diagnosis antigen in place of a single-molecule antigen [9–11]. In our previous study we constructed an epitope recombinant protein pGEX-Sj23-SjGCP and ELISA tests showed that the recombinant antigen was a promising diagnostic antigen for detecting *S. japonica* in water buffalo better than signal recombinant protein Sj23 and SjGCP [9]. In this study, we constructed two bivalent epitope recombinant antigens and two trivalent epitope recombinant antigens based on the epitope analysis of three schistosomiasis diagnosis antigens Sj23, SjPGM and SjRAD23, and parallelly compared the sensitivity and specificity of these newly constructed multi-epitope recombinant antigens with the relative single-molecule recombinant antigens, rSjPGM, rSjRAD23, rLHD-Sj23 and SEA using them as diagnostic antigens for detecting goat schistosomiasis by ELISA. The purpose of this study was to screen a promising diagnosis antigen and establish a more sensitive technique for the diagnosis of goat schistosomiasis.

## Methods

### Ethical approval

All animal care procedures were conducted in strict accordance with the Regulations for the Administration of Affairs Concerning Experimental Animals (Date issued: 1988.11.1), and all efforts were made to minimise suffering. The infection of goats and rabbits with *S. japonicum* and the sera or livers collection as well as others animal procedures were approved by the Animal Care and Use Committee of Shanghai Veterinary Research Institute, Chinese Academy of Agricultural Sciences for the use of laboratory animals (Permit ID Number: SHVRI 2013–0309).

### Animals

Male New Zealand rabbits (2.0–2.5 kg) were bought from the Shanghai Experimental Animal Center, Chinese Academy of Sciences.

### Goats sera

Ninety-one schistosome positive sera were obtained from goats artificially infected with *S. japonicum* and 44 sera from uninfected animals were collected from goats from schistosomiasis non-endemic areas. Totals of 37 *Orientobilharzia*-positive sera were collected from goats in which parasites were found in their portal venous system in Nyêmo County, XiZang and 12 *Haemonchus contortus-*positive sera were collected from goats in which parasites were found in their abomasa. Serum (10 μl) was taken from each of 20 schistosome-positive or negative sera and mixed as reference positive or negative sera, respectively. All sera were stored at −80 °C in the laboratory until use.

### Preparation of SEA

The SEA were prepared as previously described [12, 13]. Briefly, after the rabbits were infected artificially with *S. japonicum* for 42 days, the livers were collected and stored at 4 °C for one day. Then, the livers were homogenised in phosphate buffered saline (PBS). The mixture was sequentially passed through 60, 80, 120, 160, and 200 mesh screens to separate the eggs from liver tissues. The collected solution was centrifuged at 5000× *g* for 10 min. The precipitation was washed three times with PBS. The pellet was resuspended with 100 ml PBS containing 7.5 U trypsin and was incubated at 37 °C for 2 h in constant temperature shaker to remove the remaining host proteins. Then, the solution was centrifuged at 5000× *g* for 2 min and the upper liver paste was removed. The pellet was resuspended with PBS, centrifuged as previously and the upper liver paste was removed; this procedure was repeated several times until a uniform golden yellow precipitation was obtained. The qualities of the eggs were examined under the microscope. The purified eggs were diluted with suitable amounts of PBS. Freezing and thawing was repeated for three times and then sonicated using an ultrasonic cell crusher on ice. The sample was centrifuged at 12,000× *g* for 20 min. The supernatant was collected as SEA.

### Epitope analysis of SjPGM, SjRAD23 and LHD-Sj23

A series of prediction tools were used to select the epitopes of SjPGM (GenBank accession no. FN315287) and SjRAD23 (GenBank accession no. FN314619). The online software, BepiPred 1.0 was chosen to predict linear B-cell epitopes [14]. The antigenicity, surface accessibility prediction and long loops prediction were analysed by using PredictProtein and IEDB Analysis Resource [15, 16]. The T-epitope Designer and IEDB Analysis Resource were used to predict T-cell epitopes [17]. The peptides with the highest score in each software were selected. The B-cell epitopes and antigenicity of the predicted proteins were considered first. The peptides which included most high score B-cell epitopes, possessing higher antigenicity and corresponding to most or part of other prediction results were chosen as the SjPGM or SjRAD23 epitopes or epitope-rich regions.

Two epitopes were selected from SjRAD23, named as BSjRAD23-1 and BSjRAD23-2, respectively. One epitope was selected from SjPGM and named BSjPGM.

The large hydrophilic region of Sj23 (LHD-Sj23) (GenBank accession no. FN323147) was selected as a candidate fragment. Previous studies have shown that the LHD-Sj23 had a high immunogenicity and was rich region of B-cell and T-cell epitopes [18, 19]. It was also shown that the LHD-Sj23 is a good candidate antigen for detecting cattle and goat schistosomiasis [20]. The LHD-Sj23 was named BSj23.

### Construction of recombinant multi-epitope expression plasmids

The primer sets containing specific restriction enzyme sites (Table 1) were designed to amplify and fuse a desired segment against the cDNA sequence of each peptide. PCRs were performed using the cDNA of 42-day *S. japonicum* as a template. Fifty μl of reaction mixture contained 5 μl of buffer, 4 μl of 2.5 mM dNTP, 4 μl of each 10 nmol/μl primer, 0.5 μl of 5u/μl Ex Taq DNA polymerase (Takara, Japan), 2 μl of template and 34.5 μl ddH_2_O. The conditions for PCR were as follows: 95 °C for 5 min, followed by 34 cycles of 94 °C for 1 min, 55 °C for 30 s, 72 °C for 1 min, and a final extension of 72 °C for 10 min. The PCR was performed using T100™ Thermal Cycler (BIO-RAD, USA). The PCR products were purified using Mini-DNA fragment rapid purification kit (BioDev, China).

**Table 1:** Primer information for cDNA encoding multi- epitope antigens

cDNA	Primer		Sequence^a^	Enzyme
BSjRAD23-1	forward		5′-CGCGAATTCATACATTCAGGCAAGG-3′	*Eco*RI
	reverse		5′-CGCAAGCTTGGGTAGGCTAGGCT-3′	*Hin*dIII
BSj23	forward		5′-CGCGAATTCATGACTGGTGCTCTGGA-3′	*Eco*RI
	reverse		5′-CGCAAGCTTCTAGTTGCGTTTTAAG-3′	*Hin*dIII
BSjPGM-BSj23	BSjPGM	forward	5′-CGCGGATCCTGGCGTCTAAATGAAAGA-3′	*Bam*HI
		reverse	5′-CGCGAATTCAAACCAGAATGGTAGT-3′	*Eco*RI
	BSj23	forward	5′-CGCGAATTCATGACTGGTGCTCTGGA-3′	*Eco*RI
		reverse	5′-CGCAAGCTTCTAGTTGCGTTTTAAG-3′	*Hin*dIII
BSjPGM-BSjRAD23-1	BSjPGM	forward	5′-CGCGGATCCTGGCGTCTAAATGAAAGA-3′	*Bam*HI
		reverse	5′-CGCGAATTCAAACCAGAATGGTAGT-3′	*Eco*RI
	BSjRAD23-1	forward	5′-CGCGAATTCATACATTCAGGCAAGG-3′	*Eco*RI
		reverse	5′-CGCAAGCTTGGGTAGGCTAGGCT-3′	*Hin*dIII
BSjPGM-BSjRAD23-1-BSj23	BSj23	forward	5′-CGCAAGCTTATGACTGGTGCTCTGGA-3′	*Hin*dIII
		reverse	5′-CGCCTCGAGCTAGTTGCGTTTTAAG-3′	*Xho*I
BSjRAD23-2-BSjPGM-BSj23	BSjRAD23-2	forward	5′-CGCGGATCCATGGTCATACGAGCAATG-3′	*Bam*HI
		reverse	5′-CGCGAGCTCTGCGATTGGGTCTTCTGA-3′	*Sac*I
	BSjPGM	forward	5′-CGGGAGCTCTGGCGTCTAAATGAAAG-3′	*Sac*I
		reverse	5′-CGCAAGCTTAAACCAGAATGGTAG-3′	*Hin*dIII
	BSj23	forward	5′-CGCAAGCTTATGACTGGTGCTCTGGA-3′	*Hin*dIII
		reverse	5′-CGCCTCGAGCTAGTTGCGTTTTAAG-3′	*Xho*I

^a^The sequences of the restriction enzymes are underlined

The purified PCR products were digested with their respective restriction enzymes and inserted into the pET28a (+) or pET32a (+) vector (Novagen, USA). For example, to construct BSjPGM-BSj23/ pET28a (+), the nucleotide sequence of BSjPGM was digested with *Bam*HI and *Eco*RI, and inserted into pET28a (+). Then BSjPGM/pET28a (+) and the nucleotide sequence of BSj23 were digested with *Eco*RI and *Hin*dIII, then linked using T4 DNA ligase (Takara, Japan) to construct the BSjPGM-BSj23/pET28a (+). The other multi-epitope expression plasmids were constructed with the same method. All recombinant fragments were inserted into pET-28a (+) except BSj23 which was inserted into pET-32a (+). The constructed multi-epitope plasmids were transformed into *Escherichia coli* BL21 (*DE3*) (Beijing TransGen Biotech, China). All the multi-epitope plasmids were verified by PCR and restriction enzyme digestion as well as sequencing.

### Expression and purification of the recombinant antigens

*Escherichia coli* cells containing constructed plasmids were cultured in Luria-Bertani broth containing kanamycin (1 mM), in a shaker at 37 °C. Isopropyl-β-D-thiogalactopyranoside (1 Mm) was added when the OD_600_ was around 0.6 and cultures were grown for a further 6 h at 37 °C with agitation (250 rpm). Bacterial cells were then centrifuged at 12,000× *g* for 10 min and then resuspended in 10 ml PBS. Purification of recombinant proteins was applied by His•Bind Resin chromatography (Novagen, USA) following the manufacturer’s instructions. These his-tagged fusion proteins purified by affinity chromatography were analysed by sodium dodecyl sulfate-polyacrylamide gel electrophoresis (SDS-PAGE). The purified inclusion body proteins were dialyzed against PBS solution. The fusion proteins of SjPGM and SjRAD23 were obtained and purified as described above [8]. The concentration of purified recombinant proteins was measured using the BCA Protein Assay (Sangon Biotech, China).

### Comparison of the sensitivity and specificity of different recombinant antigens in goat schistosomiasis diagnosis by ELISA

Based on checkerboard titration analysis, microtiter plates (Costar, USA) were coated overnight at 4 °C with 100 μl per well containing 15 μg/ml of each tested recombinant antigen except 5 μg/ml for rBSjPGM-BSj23, or 15 μg/ml SEA diluted in carbonate bicarbonate buffer (pH 9.6). Plates were blocked with 1 % gelatin for 1 h at 37 °C. Sera at a dilution of 1:100 with PBST (PBS with 0.05 % Tween 20) was added to the wells (100 μl/well) and incubated for 1 h at 37 °C. Each serum was repeated for three wells in one test. Horseradish peroxidase-conjugated rabbit anti-goat immunoglobulin G (Santacruz, USA) diluted in 1:6000 (1:4000 for rBSjRAD23-1 and rBSjPGM-BSjRAD23-1) with PBST was added (100 μl/well) and incubated at 37 °C for 45 min. The plates were washed three times with PBST. 3,3^′^,5,5^′^- Tetramethyl benzidine dihydrochloride was added to each plates (100 μl/well) and reactions were stopped 10 min later using 2 M sulfuric acid (50 μl/well). Optical density (OD) at 450 nm was determined using a microplate reader (BioTek, USA). Each ELISA reaction was performed with a reference negative serum control and a reference positive serum control.

### Statistics

The 2.1 times of the mean absorbance value of the reference negative sera was set as the cutoff value. A sample was considered positive when its mean absorbance value was higher than the cutoff value. Statistical analysis was performed using Stata software (version 13/SE) and 95 % confidence intervals (CI) were determined for the sensitivity, specificity and cross-reactivity of each test. Sensitivity and specificity were calculated as follows: sensitivity = no. of true positives/(no. of true positives + no. of false negatives); specificity = no. of true negatives/(no. of true negatives + no. of false positives). Sensitivity, specificity and cross-reactivity for recombinant proteins were compared with that of SEA.

## Results

### Selection and cloning of cDNA encoding epitope-rich peptides from SjPGM and SjRAD23

**Fig. 1:**
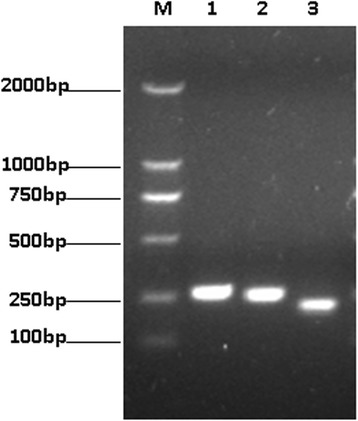
The nucleic acid fragments of BSjPGM, BSjRAD23-1 and BSjRAD23-2 by PCR. Lane M: Marker DL 2000 DNA Ladder; Lane 1: BSjPGM; Lane 2: BSjRAD23-1; Lane 3: BSjRAD23-2

Taking all of the analysis results from each predictive tool into consideration (Table 2), one polypeptide was selected from SjPGM and named BSjPGM, and two polypeptides were selected from SjRAD23 and named BSjRAD23-1 and BSjRAD23-2. BSjPGM contained 82 amino acids encoding the 85 to 166 amino acid sequence of SjPGM with two B-cell epitopes and two T-cell epitopes. BSjRAD23-1 and BSjRAD23-2 contained 78 amino acids and 65 amino acids, encoding amino acids 46–123 and 166–230 of the sequence of SjRAD23, with one and two B-cell epitopes as well as one and two T-cell epitopes respectively. The nucleotide sequence encoding BSjPGM, BSjRAD23-1 and BSjRAD23-2 were amplified (Fig. 1), sequenced and sub-cloned into pET-28a (+).

**Table 2:** Prediction results using online software for SjPGM and SjRAD23

Online software		Application	Results (high score peptides)
BepiPred 1.0	SjPGM	B-cell epitopes prediction	33–39, 101–109, 119–138
	SjRAD23		76–123, 174–178, 194–230
IEDB Analysis	SjPGM	B-cell epitopes prediction	29–40, 98–110, 119–138, 197–203
	SjRAD23		51–59, 75–130, 173–180, 194–230
IEDB Analysis	SjPGM	Antigenicity scale	28–38, 93–98, 109–115, 119–129, 139–167, 221–243
	SjRAD23		42–49, 58–77, 101–128, 163–169, 192–238, 309–330
T-epitope Designer	SjPGM	T-epitope prediction	115–123, 132–140
	SjRAD23		59–67, 181–189, 195–203
PredictProtein	SjPGM	Long loops prediction	23–32, 91–100, 116–152
	SjRAD23		34–43, 51–65, 72–162, 182–230, 282–305
IEDB Analysis	SjPGM	Accessibility prediction	82–91, 98–104, 114–122
	SjRAD23		52–63, 91–101, 107–121, 175–180, 197–203

### Construction of recombinant multi-epitope expression plasmids

**Fig. 2:**
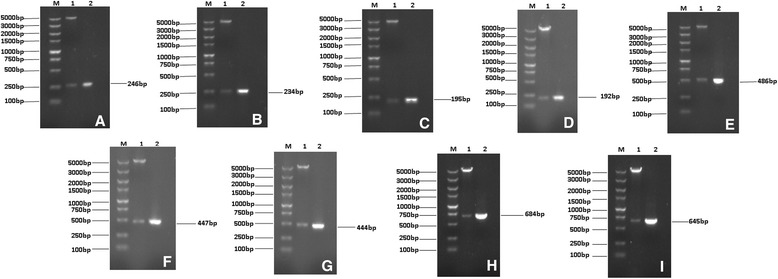
Analysis of the recombinant plasmids by restriction enzyme digestion and PCR. **a**, pET-28a (+)-BSjPGM; **b**, pET-28a (+)-BSjRAD23-1; **c**, pET-28a (+)-BSjRAD23-2; **d**, pET-32a (+)-BSj23; **e**, pET-28a (+)-BSjPGM-BSjRAD23-1; **f**, pET-28a (+)-BSjRAD23-2-BSjPGM; **g**, pET-28a (+)-BSjPGM-BSj23; **h**, pET-28a (+)-BSjPGM-BSjRAD23-1-BSj23; **i**, pET-28a (+)-BSjRAD23-2-BSjPGM-BSj23. Lane 1: The recombinant plasmid digested with restriction enzymes; Lane 2: PCR results using the recombinant plasmid as template; Lane M: Marker DL5000 DNA Ladder

A total of nine expression plasmids were successfully constructed: BSjPGM/pET-28a (+); BSjRAD23-1/pET-28a (+): BSjRAD23-2/pET-28a (+); BSj23/pET-32a (+); BSjPGM-BSj23/pET-28a(+); BSjPGM-BSjRAD23-1/pET-28a (+); BSjPGM-BSjRAD23-1-BSj23/pET-28a (+); BSjRAD23-2-BSjPGM-BSj23/pET-28a(+); and BSjRAD23-2-BSjPGM/pET-28a(+). The SjPGM/pET-28a (+) and SjRAD23/pET-28a (+) plasmids were constructed according to a previous study [6]. The successful construction of recombinant multi-epitope plasmids were verified by PCR and sequenced (Fig. 2).

### Expression and purification of the recombinant proteins

**Fig. 3:**
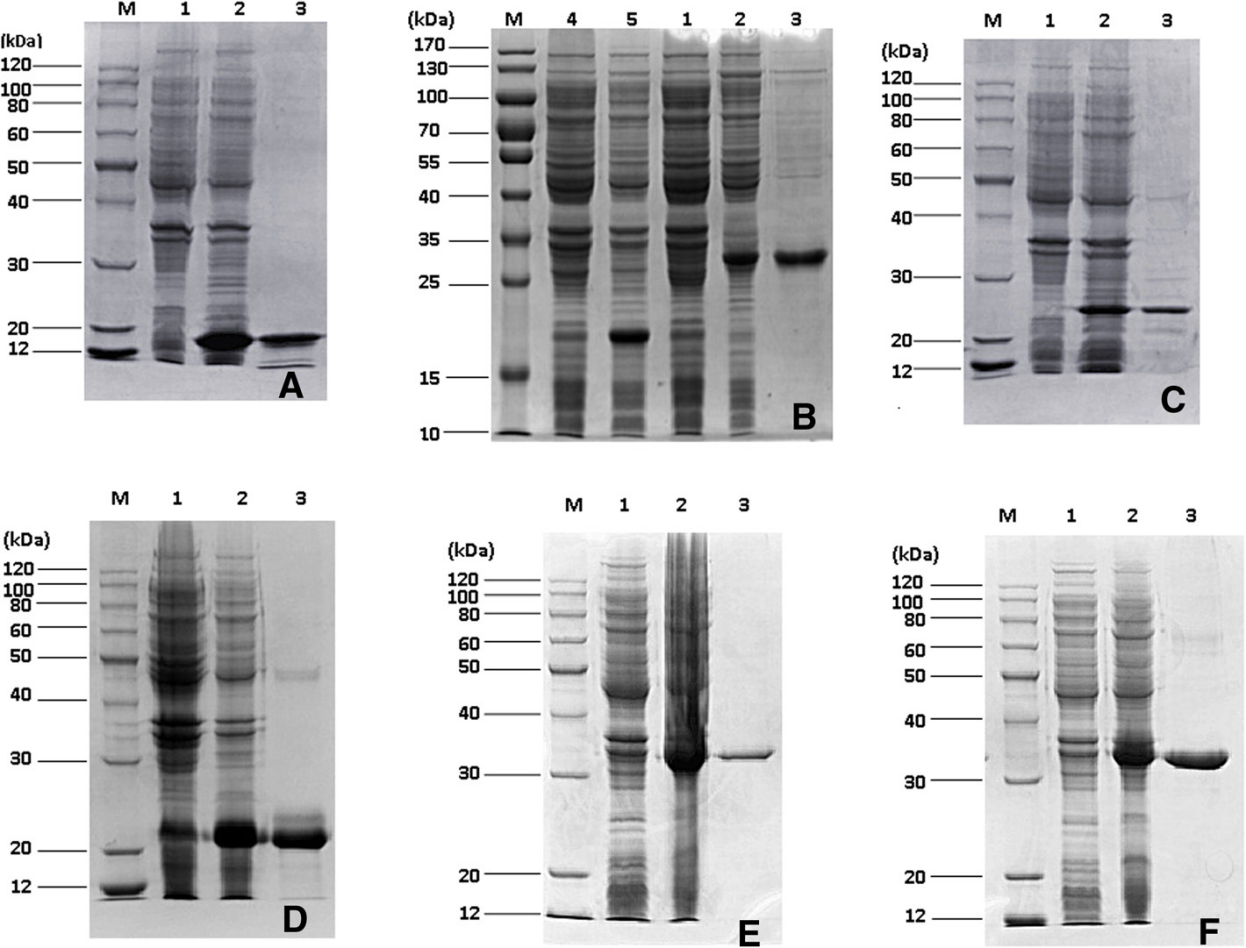
Expression and purification analysis of recombinant proteins. **a**, rBSjRAD23-1; **b**, rBSj23; **c**, rBSjPGM-BSjRAD23-1; **d**, rBSjPGM-BSj23; **e**, rBSjPGM-BSjRAD23-1-BSj23; **f**, rBSjRAD23-2-BSjPGM-BSj23. Lane M: Molecular markers; Lane 1: Total extract from vectors pET-28a (+) (B: pET-32a (+) vector with no IPTG) after induction with 1 mM IPTG; Lane 2: Total extract from recombination proteins after induction with 1 mM IPTG; Lane 3: The recombinant protein purified through Ni^2+^-charged column chromatography; Lane 4: Total extract from pET-32a (+) vector; Lane 5: Total extract from from pET-32a (+) vector after induction with 1 mM IPTG

All of the recombinant expression plasmids were successfully transformed into *E. coli* BL21 (*DE3*) and six kinds of crude fusion proteins were successfully expressed except rBSjPGM, rBSjRAD23-2 and rBSjRAD23-2-BSjPGM. The recombinant antigens, rSjPGM, rSjRAD23, rBSjPGM-BSjRAD23-1 and rBSjRAD23-1, were expressed in soluble form. The others existed as the form of inclusion body proteins which were dissolved in 8 M urea solution. The expected molecular mass and the sodium dodecyl sulfate-polyacrylamide gel electrophoresis (SDS-PAGE) of the six recombinant proteins are shown in Table 3 and Fig. 3.

**Table 3:** Expected sizes of the recombinant proteins

The multi-epitope antigens	Number of amino acids	Molecular mass (kDa)
rBSjRAD23-1	78	16
rBSj23	64	7
rBSjPGM-BSjRAD23-1	162	25.8
rBSjPGM-BSj23	148	22.6
rBSjPGM-BSjRAD23-1-BSj23	228	34
rBSjRAD23-2-BSjPGM-BSj23	215	32

### Diagnosis of goat schistosomiasis using recombinant antigens as detecting antigens

**Fig. 4:**
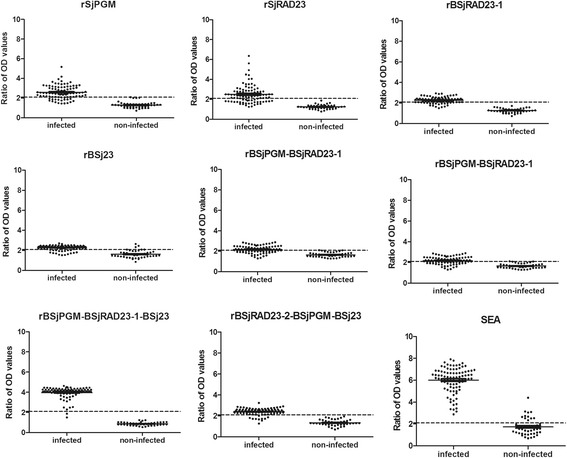
Analysis of the recombinant proteins and SEA for the diagnosis of goat schistosomiasis by ELISA. Sera from 91 schistosome infected goats and 44 uninfected goats were tested

To assess the potential of rLHD-Sj23, rSjPGM, rSjRAD23 and the newly constructed multi-epitope proteins as diagnostic antigens for goat schistosomiasis, sera from 91 schistosome-infected goats, 44 non-infected goats, 12 goats infected with *H.contortus* and 37 *Orientobilharzia*-infected goats were tested using these recombinant antigens and SEA (control) as detecting antigen, yielding results shown in Fig. 4 and Table 4. Among the eight recombinant antigens, three showed higher sensitivity with 89.01 % (81/91, 95 % CI: 80.72–94.60 %) for rBSjRAD23-2-BSjPGM-BSj23, 93.41 % (86/91, 95 % CI: 87.64–98.19 %) for rBSjPGM-BSj23 and 97.80 % (89/91, 95 % CI: 92.29–99.73 %) for rBSjPGM-BSjRAD23-1-BSj23. Most of the multi-epitope antigens showed higher sensitivity than single molecular recombinant antigens rLHD-Sj23, rSjRAD23 and rSjPGM except for rBSjPGM-BSjRAD23-1 (59.34 %, 95 % CI: 48.53–69.52 %). The sensitivity of rBSjPGM-BSjRAD23-1-BSj23 (97.8 %) was higher than the other recombinant antigens. The sensitivity of all recombinant antigens was lower than that of SEA (100.0 %, 95 % CI: 96.03–100 %).

**Table 4:** Comparison of the sensitivity and specificity of various recombinant antigens in the diagnosis of goat schistosomiasis by ELISA

Sera sample		*S. japonicum*-infected goats	Uninfected goats	*H. contortus*-infected goats	*Orientobilharzia* -infected goats
No. of cases		91	44	12	37
rSjPGM	No. of positive	72	0	0	0
	Positive rate (%)	79.12	0	0	0
rSjRAD23	No. of positive	60	0	0	0
	Positive rate (%)	65.93	0	0	0
rBSjRAD23-1	No. of positive	67	0	0	4
	Positive rate (%)	73.63	0	0	10.82
rBSj23	No. of positive	73	3	0	2
	Positive rate (%)	80.22	6.82	0	5.41
rBSjPGM-BSjRAD23-1	No. of positive	54	1	0	0
	Positive rate (%)	59.34	2.27	0	0
rBSjPGM-BSj23	No. of positive	86	0	0	1
	Positive rate (%)	93.41	0	0	2.70
rBSjPGM-BSjRAD23-1-BSj23	No. of positive	89	0	1	5
	Positive rate (%)	97.80	0	8.33	13.51
rBSjRAD23-2-BSjPGM-BSj23	No. of positive	81	0	0	0
	Positive rate (%)	89.01	0	0	0
SEA	No. of positive	91	11	3	31
	Positive rate (%)	100.00	25.00	25.00	83.78

The specificity of most of the recombinant antigens was 100 % (44/44, 95 % CI: 91.96–100 %) except rBSjPGM-BSjRAD23-1 (97.73 %, 43/44, 95 % CI: 87.98–99.94 %) and rBSj23 (93.18 %, 41/44, 95 % CI: 81.34–98.57 %) which all were higher than that of SEA (72 %, 33/44, 95 % CI: 59.66–86.81 %) (Table 4). These antigens were also used to detect sera from *Orientobilharzia*-infected goats and *H. contortus*-exposed goats. No cross-reactivity with sera from *H. contortus*-exposed goats was observed for most of the recombinant antigens tested except for rSjBPGM-BRAD23-1-BSj23 (8.33 %, 1/12, 95 % CI: 2.11–38.48 %) compared with that of SEA (25 %, 3/12, 95 % CI: 5.49–57.91 %) (Table 4). Four recombinant antigens (rSjPGM, rSjRAD23, rBSjRAD23-2-BSjPGM-BSj23 and rBSjPGM-BSjRAD23-1) have no cross-reactivity with sera from *Orientobilharzia*-infected goats. The cross-reactivities with orientobilharziasis of other recombinant antigens were 10.82 % (rBSjRAD23-1, 4/37, 95 % CI: 3.03–25.42 %), 5.41 % (rBSj23, 2/37, 95 % CI: 0.66–18.19 %), 2.70 % (rBSjPGM-BSj23, 1/37, 95 % CI: 0.07–14.16 %) and 13.51 % (rBSjPGM-BSjRAD23-1-BSj23, 5/37, 95 % CI: 4.54–28.77 %), respectively. As a control, SEA showed a very high cross-reactivity (31/37, 83.78 %, 95 % CI: 67.99–93.81 %) with sera from *Orientobilharzia*-infected goats.

## Discussion

It is still a huge challenge to diagnose schistosomiasis in low endemic areas in China after six decades of integrated schistosomiasis control [21, 22]. Animal surveillance is a loophole in the transmission of schistosomiasis as there is a lack of importance given to the role of animal hosts [2], especially goats in some endemic regions. As confirmed in previous studies, the sedimentation technique and Kato-Katz or hatching test may not detect light infections in reservoir hosts which have undergone mass drug administration [23–25]. Antibody-detection has been shown to be more sensitive than parasitological techniques and is needed in areas characterised by a low level of transmission, low prevalence and particular low intensity [22, 26]. Although serological diagnosis is not able to discriminate between active and past infections and has higher cross reactions with other parasites [7], many methods based on the detection of antigens or specific antibodies have been developed and applied in the field. Some immunological tests developed in China have acceptable performance characteristics and some serological methods are used largely as a screening tool in China, such as indirect hemagglutination assay (IHA) [27, 28]. Molecular methods, mainly DNA amplification techniques by PCR, based on the genomic DNA of schistosomes are able to detect DNA from all phases of the life-cycle of this parasite [29, 30]. Although the adequacy and sensitivity of the PCR methods have been proven in schistosome infections in water buffaloes [31] and in other helminth and protozoan infections in humans [32, 33], the sensitivity of molecular methods can be affected by the degree of infection and methods have poor agreement [34]. Furthermore, the molecular diagnostic techniques are more expensive than serological diagnostic methods.

Schistosome-infected domestic animals are an important source for the transmission of schistosomiasis [35]. Goats are a source of infection due to the large population sizes, wide range of activities and high infection rates in some schistosomiasis endemic regions. The role of goats in the spread of schistosomiasis may become increasingly prominent and could be a potential hazard, causing a rebound of schistosomiasis in snail habitats if there are no diagnosis measures as in the effective control on infected bovines, patients and their waste [36]. The detection for goat schistosomiasis should warrant further attention and establishing a sensitive and specific diagnostic test is urgently required.

The LHD-Sj23, SjPGM and SjRAD23 were selected to test in this study because they possess higher sensitivity and specificity in detecting water buffalo schistosomiasis [8, 20]. But when we used these single recombinant molecules as diagnosis antigen to detect goat schistosomiasis, although they all had higher specificity, the sensitivity of the ELISA for rSjPGM and rSjRAD23 were only 79.12 and 65.93 %, lower than the 91.35 % (95/104) and 88.46 % (92/104) for an ELISA with the same antigens in water buffalo; this may correlate with species or immune responses difference between goat and water buffalo.

To further increase the sensitivity of the diagnostic technique for goat schistosomiasis, epitopes of SjPGM and SjRAD23 were predicted. Recombinant multi-epitope proteins were constructed and their potential as diagnostic antigens for goat schistosomiasis was evaluated in this study. To enhance the accuracy of epitope prediction, several online software analytical methods were applied. The peptides with the highest score in each online method were selected. The peptides that included B-cell epitope-rich regions and possessed higher antigenicity were considered first. According to the conclusion that synthetic peptides containing linear-cell epitopes can be used to raise antibodies against specific proteins and as a diagnostic reagent. B-cell epitopes are the regions by which some proteins or antibodies (produced by B-cells) bind [37]. In the end, two polypeptides (76–123, 194–230) from SjRAD23 and one polypeptide (85–166) from SjPGM were selected. Considering that the LHD-Sj23 is a B-cell epitope-rich region and has been confirmed to be a sensitive diagnostic molecule for goat and cattle schistosomiasis [18–20], three newly predicted polypeptides and the LHD-Sj23 were chosen to construct the recombinant multi-epitope protein expression plasmids. A total of five recombinant plasmids were constructed with four of them being successfully expressed.

Four recombinant proteins, four recombinant multi-epitope proteins and SEA were applied as diagnostic antigens to detect goat schistosomiasis. The results show that the sensitivity of three recombinant multi-epitope proteins, except for rBSjPGM-BSjRAD23-1, was higher than those of four single-molecule recombinant proteins. Among the three recombinant multi-epitope antigens, rBSjPGM-BSjRAD23-1-BSj23 exhibited the highest sensitivity (97.8 %), slightly lower than SEA (100 %), similarly, the specificity of rBSjPGM-BSjRAD23-1-BSj23 was 100 %, significantly higher than that of SEA (75 %). In addition, the cross-reactivity with orientobilharziasis and haemonchosis of rBSjPGM-BSjRAD23-1-BSj23 was 13.51 and 8.33 %, also significantly lower than that of SEA (83.78 and 25 %). The other two recombinant multi-epitope proteins with higher sensitivity and specificity in diagnosis of goat schistosomiasis were rBSjPGM-BSj23 (93.4 and 100 %) and rBSjRAD23-2-BSjPGM-BSj23 (89 and 100 %), both also showing lower cross-reactivity with orientobilharziasis (2.7 % for rBSjPGM-BSj23 and 0 % for rBSjRAD23-2-BSjPGM-BSj23) and haemonchosis (0 % for both). The conclusion that constructing multi-epitope antigens can increase the sensitivity of serological detection methods has also been reported in *Toxoplasma gondii* [10, 11]. All above-mentioned show constructing multi-epitope antigens is a worthwhile way.

Compared with the results obtained from SEA, which has been widely employed as the diagnostic antigen in most developed serological detecting methods for schistosomiasis, the application of recombinant antigens as diagnosis antigens significantly increased the specificity of diagnostic techniques in this study. It will help to accurately grasp the epidemic, to assess the effect of drug treatment or prevention, and has great significance in reducing repeated drug use for false-positive animals. The recombinant antigens were convenient to prepare, inexpensive and performed favorably to standardise diagnostic techniques, they therefore have good prospects.

Among the three polypeptides which possessed epitope-rich regions selected from SjPGM and SjRAD23, only BSjRAD23-1 was successfully expressed in *E. coli* in this study. The ELISA tests showed that the sensitivity of rBSjRAD23-1 (73.63 %) was higher than that of rSjRAD23 (65.93 %), and rBSjPGM-BSjRAD23-1-BSj23 (97.80 %) was higher than that of rBSjPGM-BSj23 (86 %), suggested that recombinant rBSjRAD23-1 possessed potential value as a goat schistosomiasis diagnosis antigen. The recombinant LHD-Sj23 has proven to be a good diagnostic antigen for schistosomiasis in some previous reports [20, 38]. In this study, we also noted that the inclusion of the LHD-Sj23 peptide in the recombinant multi-epitope antigens could increase the sensitivity in the diagnosis of goat schistosomiasis. The three top sensitive antigens (rBSjPGM-BSjRAD23-1-BSj23, 97 %; rBSjPGM-BSj23, 93.41 %; rBSjRAD23-2-BSjPGM-BSj23, 89.01 %) all possessed the composition of the LHD-Sj23 peptide.

The World Health Organisation has noted that case detection will be a problem when elimination of the disease is at hand [39]. Goats are an important host of *S. japonicum* and should be involved in animal monitoring system in some endemic areas in China. The study presented here suggests that the application of recombinant multi-epitope proteins may increase the sensitivity of diagnostic techniques for schistosomiasis, and that the recombinant antigen rBSjPGM-BSjRAD23-1-BSj23 has the potential to be used as a diagnostic antigen for goat schistosomiasis. More sera samples including schistosome-infected and uninfected goats, as well as other parasite-infected goats sera are needed to perform and confirm this result, especially sera from schistosomiasis in low endemic areas and from animals with low infection intensity.

## Conclusion

In conclusion, three recombinant multi-epitope antigens rBSjPGM-BSj23, rBSjRAD23-2-BSjPGM-BSj23 and rBSjPGM-BSjRAD23-1-BSj23 exhibited higher sensitivity and higher specificity than the four recombinant single molecules, as well as higher specificity and lower cross-reactivity than SEA. Among all of the tested recombinant antigens, rBSjPGM-BSjRAD23-1-BSj23 showed the highest sensitivity (97.8 %) and 100 % specificity, having the potential to be used as a diagnostic antigen for goat schistosomiasis. The study also suggested that the application of recombinant multi-epitope diagnostic antigens may increase the sensitivity of the diagnosis technique.
